# Mid-infrared ultra-high-*Q* resonators based on fluoride crystalline materials

**DOI:** 10.1038/ncomms13383

**Published:** 2016-11-21

**Authors:** C. Lecaplain, C. Javerzac-Galy, M. L. Gorodetsky, T. J. Kippenberg

**Affiliations:** 1 École Polytechnique Fédérale de Lausanne (EPFL), CH-1015 Lausanne, Switzerland; 2 Russian Quantum Center, 143025 Skolkovo, Russia; 3 Faculty of Physics, M.V. Lomonosov Moscow State University, 119991 Moscow, Russia

## Abstract

The unavailability of highly transparent materials in the mid-infrared has been the main limitation in the development of ultra-sensitive molecular sensors or cavity-based spectroscopy applications. Whispering gallery mode microresonators have attained ultra-high-quality (*Q*) factor resonances in the near-infrared and visible. Here we report ultra-high *Q* factors in the mid-infrared using polished alkaline earth metal fluoride crystals. Using an uncoated chalcogenide tapered fibre as a high-ideality coupler in the mid-infrared, we study via cavity ringdown technique the losses of BaF_2_, CaF_2_, MgF_2_ and SrF_2_ microresonators. We show that MgF_2_ is limited by multiphonon absorption by studying the temperature dependence of the *Q* factor. In contrast, in SrF_2_ and BaF_2_ the lower multiphonon absorption leads to ultra-high *Q* factors at 4.5 μm. These values correspond to an optical finesse of 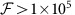, the highest value achieved for any type of mid-infrared resonator to date.

## Abstract (web summary)

Highly sensitive trace-gas detection is possible in the mid-infrared range with transparent microresonators. Here, the authors directly measure the necessary ultra-high quality factors of microresonators made from fluoride crystal materials using a tapered chalcogenide fibre.

## Graphical abstract

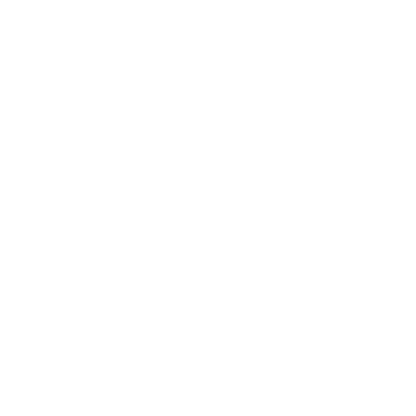

The mid-infrared (mid-IR) spectral window 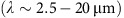 is a very useful range for spectroscopy, chemical and biological sensing, materials science and industry as it includes strong rotational–vibrational absorption lines of many molecules, as well as two atmospheric transmission windows of 3–5 and 8–13 μm. In this band, the absorption strengths of molecular transitions are typically 10 to 1,000 times greater than those in the visible or near-infrared (near-IR), offering the potential to identify the presence of substances with extremely high sensitivity and selectivity, as required in trace gas detection^1^,^2^,^3^, breath analysis^4^,^5^ and pharmaceutical process monitoring. The invention of the quantum cascade laser (QCL)^6^ was a scientific and technological milestone in the development of mid-IR laser sources. Today, continuous-wave QCLs operate at room temperature, offer high output power (watts) and are a commercial technology^7^. These features lead to their wide adoption for spectroscopic applications in the mid-IR region. However, their free running linewidth is subject to undesired noise that makes their frequency stabilization challenging^8^. High finesse optical cavities have the potential for substantial reduction of laser noise^9^,^10^ and have been recently investigated^11^.

The exploration of ultra-high-quality (*Q*) cavities in the mid-IR is a relatively new area driven by the development of QCL frequency stabilization^11^, ultra-sensitive molecular sensors^12^, cavity-based spectroscopy^4^,^13^ and optical frequency combs^14^. ZBLAN^15^ and chalcogenide (ChG) glass^16^ resonators also appear as promising for mid-IR wavelengths. Moreover, the silicon platform^17^ has enabled the fabrication of mid-IR microresonators at 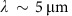. To date, *Q* factors of 10^5^ are already achieved in silicon-on-sapphire^18^ and ChG glass-on-silicon^19^, whereas *Q* factors of 10^4^ are obtained in silicon-on-calcium fluoride^20^. Yet to date, achieving ultra-high *Q* factors in the mid-IR (or correspondingly high optical finesse) is an outstanding challenge. In the near-IR, high finesse is achieved using Fabry–Pérot cavities based on supermirrors^21^ or using microcavities based on whispering gallery mode (WGM). Interference coatings based on substrate-transferred single-crystal multilayers show the potential for ≤100 p.p.m. of optical losses in the mid-IR (potentially out to 7 μm)^22^.

Owing to high purity and low material losses, crystalline WGM microresonators^23^ exhibit the highest *Q* factors in the near-IR and visible spectral ranges (along with the highest reported optical finesse of 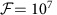 as in ref. 24). Ultra-high *Q* factors (>10^8^) are routinely obtained in magnesium fluoride (MgF_2_) and calcium fluoride (CaF_2_)^25^,^26^, and can be as high as 10^11^ in CaF_2_ (ref. 24). Recently, soft fluoride crystals such as barium fluoride (BaF_2_) and strontium fluoride (SrF_2_) have equally demonstrated ultra-high *Q* in the near-IR^27^,^28^. Such ultra-high *Q* factors have allowed for the generation of Kerr combs^23^, as well as temporal dissipative soliton formation^29^,^30^ at low (mW) power levels, and enabled low phase noise microwave generation^23^, as well as ultra narrow-linewidth lasers^10^. However, the mid-IR is a largely unchartered territory in what concerns ultra-high-*Q* optical microresonators and material properties have not been investigated thoroughly. There have been several recent studies investigating mid-IR properties of the important class of crystalline microresonators^11^,^31^,^32^. Using QCLs, recent work has reported *Q* factors of ∼10^7^ at 4.5 μm in MgF_2_ (ref. 31), CaF_2_ (refs 11, 31, 32) and BaF_2_ microresonators^31^, which is more than two orders of magnitude lower than the near-IR values. Unfortunately, the challenges in measuring *Q* factors have led to some nonphysical values being reported in literature, that is, exceeding the theoretical limit imposed by multiphonon absorption at this wavelength^32^. Therefore, it is of utmost importance to provide an authoritative and accurate account of the mid-IR quality factors above 4 μm—the range where QCLs operate to guide future experiments in this wavelength range.

Here we study systematically the optical *Q* factors of four crystalline materials transparent in the mid-IR spectrum from the alkaline earth metal fluoride *X*F_2_ family (where *X*=Ca, Mg, Ba and Sr). Note that other combinations such as *X*=Be and Ra are water soluble or toxic and therefore not suitable. We directly compare the *Q* factor at 1,555, 2,000, 3,000 and 4,500 nm and, importantly, observe that the intrinsic material absorption in MgF_2_ poses a limit to the *Q* factor, which is greatly reduced in BaF_2_ and SrF_2_. This demonstrates that material absorption limits the *Q* factor in this class of microresonators, because in the visible and near-IR spectral ranges the intrinsic absorption is many orders of magnitude lower.

To measure accurately the losses of crystalline materials in the mid-IR, we developed an efficient coupling technique based on an optical tapered fibre made out of ChG glass. It allows for light from a QCL to be evanescently coupled to a crystalline microresonator via a ChG-uncoated tapered fibre. We show that critical coupling^33^ is achieved with high ideality^34^, necessary for faithful *Q* factor measurements, and extend this technique to the mid-IR. We measured a critically coupled factor of *Q*_c_∼1 × 10^7^ for a MgF_2_ microresonator, a value at the theoretical limit of multiphonon absorption at this wavelength^31^. To further verify the intrinsic multiphonon behaviour of MgF_2_, we undertook temperature-dependence measurements. Theory predicts that MgF_2_ microresonators that are material loss limited, should exhibit a temperature dependent *Q* factor^35^, and the data presented here demonstrate the temperature variation of the multiphonon losses. This variation is consistently not observed with SrF_2_ or BaF_2_, which are not limited by this process. Using a cavity ringdown method, we demonstrate ultra-high-*Q* mid-IR resonances. This is only possible with the proper choice of crystals, which opposite to previous work^32^, implies not using MgF_2_ but rather BaF_2_ or SrF_2_. The measured mid-IR resonances feature ultra-high intrinsic factors of *Q*_0_≥6 × 10^8^ at 4.4 μm (more than a 10-fold improvement compared with previous results), exhibiting the highest observed finesse of 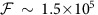 for any cavity in the mid-IR so far. These losses, when expressed as mirror losses in an equivalent Fabry–Pérot cavity, correspond to a mirror loss of ∼50 p.p.m. at 4.4 μm.

## Results

### Mid-IR crystalline microresonators properties and fabrication

**Figure 1:**
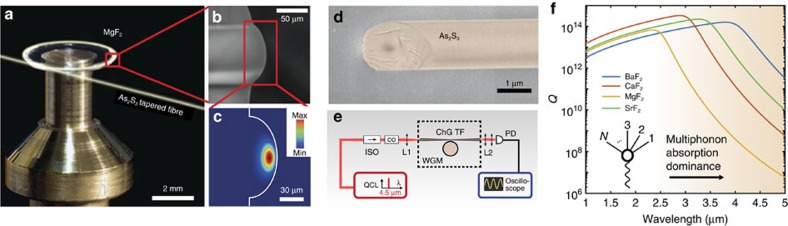
Mid-IR crystalline microresonators and uncoated ChG tapered fibre. (**a**) MgF_2_ crystalline microresonator with a diameter of ∼5 mm. WGMs of the microresonator are excited via evanescent coupling using a ChG, that is, ChG (As_2_S_3_) tapered fibre. (**b**) Scanning electron microscope (SEM) image of the MgF_2_ protrusion. Its radius of curvature, which confines the mode in the azimuthal direction, is ∼50 μm. (**c**) Finite element model simulations of the optical intensity profile of the fundamental WGM at 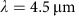. (**d**) SEM image of the waist of a ChG tapered fibre with subwavelength diameter of 1.2 μm. (**e**) Experimental set-up composed of a WGM microresonator pumped by a QCL evanescently coupled through a ChG tapered fibre (ChG TF), followed by an oscilloscope to record the transmission. An optical isolator (ISO) protects the pump laser from Fresnel reflection (∼14%) at the cleaved fibre ends. Mid-IR free space control optics (CO), including waveplates, neutral densities and a mid-IR electro-optic modulator. L1 and L2 are lenses for free space coupling into the ChG TF. PD, photodetector. Tapered fibre and microresonator are kept under a dry and inert atmosphere to preserve from degradation and physical ageing of ChG fibre^43^. (**f**) Quality factor dependence of different fluoride crystals with respect to the wavelength. For mid-IR wavelengths, multiphonon absorption competes with Rayleigh scattering and strongly impacts the *Q* factor. The orange shading highlights increasing multiphonon absorption contribution. The inset depicts a *N*-phonon creation process within the multiphonon absorption of one photon.

We are interested in crystalline materials whose transparency window includes the region of 3–5 μm, a range, where QCL are commercially available. We studied four different crystalline materials such as MgF_2_, CaF_2_, BaF_2_ and SrF_2_, which are available commercially with high purity (and used, for example, for deep ultraviolet lenses). These crystals feature ultra-high *Q* in the near-IR^25^,^26^,^27^,^28^ and anomalous group velocity dispersion in the mid-IR, a requirement for Kerr comb and (bright) temporal soliton formation based on the Kerr nonlinearity. However, the theoretically predicted limiting loss mechanism of the *Q* factor in the mid-IR results from multiphonon absorption processes^36^,^37^,^38^, involving the coupling of the incident light with fundamental molecular vibrational modes of the material, a loss mechanism not observed so far in the near-IR in such crystals. Usually this reduces the mid-IR cutoff wavelength to a much shorter value than that of the transparency window's maximum wavelength. In the multiphonon-dominated regime the absorption coefficient is given by 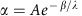 and largely surpasses any Rayleigh scattering contribution that is proportional to 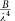, where *A*, 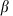 and *B* are materials properties^38^. Therefore, the *Q* factor limit is given by 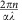, where *n* is the refractive index and 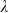 the wavelength. Figure 1f presents the dependence of the *Q* factor on the wavelength for alkaline earth metal fluoride crystalline materials^39^. It reveals that even though a material displays low Rayleigh scattering and ultra-high *Q* in the near-IR, it will be subject to multiphonon absorption in the mid-IR that increases by ∼2 — 6 orders of magnitude at 4.5 μm compared with 1.5 μm for these crystal families. On the basis of theoretical considerations alone, BaF_2_ and SrF_2_ seem ideal candidates to achieve ultra-high *Q* in the mid-IR in comparison with MgF_2_ due to lower multiphonon absorption. Though there has been considerable interest in highly transparent infrared materials for the last decades, only spectrophotometric measurements were carried out to study absorption due to technical limitations of using microresonators in the mid-IR^36^,^37^,^38^. We emphasize that conventional methods (for example, Fourier transform infrared spectroscopy) to investigate loss are not suitable to probe the losses at the level of p.p.m., as required to achieve ultra-high *Q*.

We fabricated our microresonators either from disk or cylinder blanks. The microresonators were first shaped by grinding or diamond-cutting tool and polished in an air-bearing spindle by successive smaller diamond particle slurries to obtain a smooth protrusion as visualized in Fig. 1b. Their final diameters are ∼5 mm. We analysed the microresonators in a scanning electron microscope, and from this we determined the transverse radii of the crystalline MgF_2_ disk to be of 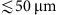. The intensity profile of the fundamental WGM of the MgF_2_ microresonator in the mid-IR is displayed in Fig. 1c. From finite element model simulations we obtained an effective mode area of *A*_eff_∼600 μm^2^ at 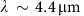. To ensure that we are not limited by scattering losses from residual surface roughness, optical quality factors were first measured at 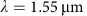, with a [Fig f2][Fig f3][Fig f4]tunable, narrow-linewidth (short-term <100 kHz) fibre laser using silica tapered fibres to excite the WGM^26^. The MgF_2_ disk features loaded optical factors of *Q*≥5 × 10^8^; CaF_2_ and SrF_2_ exhibit *Q* factors of ∼2 × 10^9^ and the BaF_2_ cylinder features *Q*∼6.4 × 10^9^ (detailed below and in Fig. 5).

### ChG tapered fibres as high-ideality mid-IR couplers

**Figure 2 f2:**
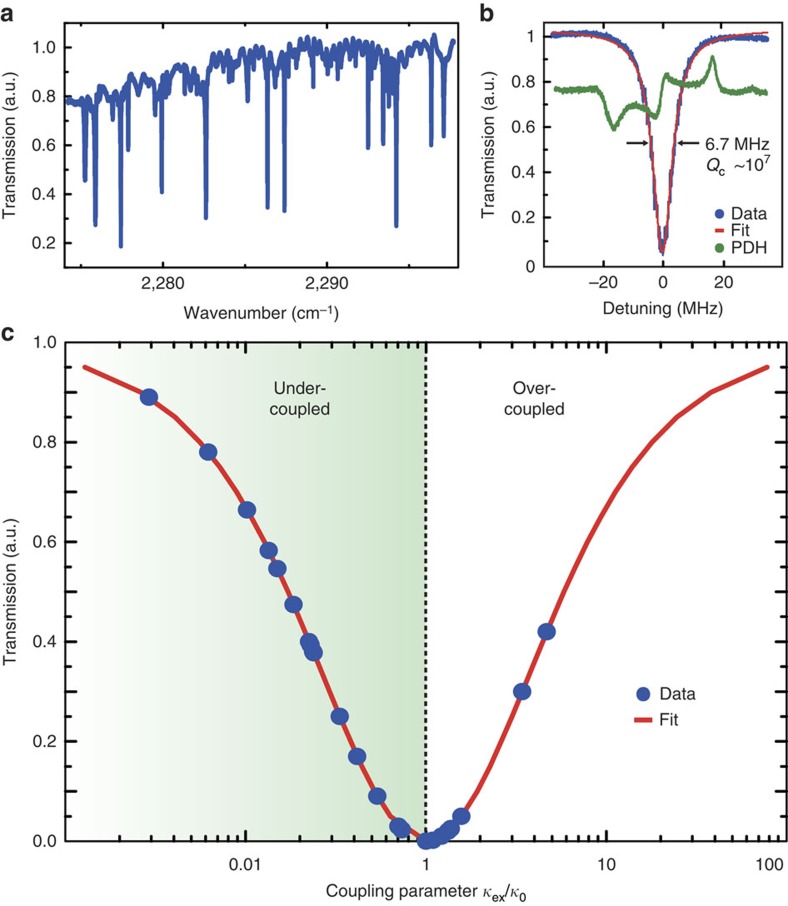
Chalcogenide taper coupling efficiency around 4.5 μm (**a**) Representative transmission spectrum composed of several resonance families over a wide mid-IR range when taper–resonator coupling is achieved with the MgF_2_ microresonator and set-up described in Fig. 1. (**b**) Measurement (blue curve) of a resonance linewidth at critical coupling with frequency calibration provided by a Pound–Drever–Hall (PDH) signal (green curve) and Lorentzian fit (red curve). The typical full-width at half maximum width of 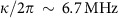 corresponds to a critically coupled quality factor of *Q*_c_∼1.0 × 10^7^. (**c**) Transmission as a function of the coupling parameter 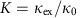 for varying taper waist radius. The dashed line marks the critical coupling point 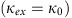. The experimental data (blue circles) are consistent with the theoretical model 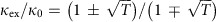 (red curve) demonstrating that the ChG taper behaves as a nearly ideal coupler in mid-IR with close to unity ideality.

To measure far in the infrared, we used a mode-hop-free tunable QCL (Daylight Solutions Inc.). To couple QCL light into the microresonator we developed uncoated optical tapered fibres made out of ChG glass^40^,^41^. ChG fibres are particularly attractive due to their low loss in the mid-IR^42^ and commercial availability. However, they were never used previously as a low-loss, uncoated tapered optical waveguide because of technical fabrication challenges in their tapering process and maintenance, due to the low melting point of ChG glasses (∼200 °C for As_2_S_3_). We have successfully developed a tapering set-up with a feedback controlled electrical heater to adiabatically pull low-loss uncoated tapered ChG fibres. To verify the theoretically expected taper waist, scanning electron microscope images were taken to optimize the tapering process. The ChG (As_2_S_3_) tapered fibre was fabricated from a commercially available, IRflex IRF-S 9 nonlinear mid-IR fibre, made from extra-high-purity ChG glass (see Methods section). The fibre is transparent from 1.5 to 6.5 μm and has a core diameter of 9 μm and a high nonlinear refractive index of *n*_2_=2.7. The ChG tapered fibre was pulled down to a diameter to phase match the fundamental WGM. For instance, for a MgF_2_ microresonator radius of *R*∼2.5 mm, the optimum taper waist in the mid-IR corresponds to a subwavelength diameter around 1 μm (Fig. 1d). The experimental set-up is described in Fig. 1e. The pump laser is a 200 mW continuous wave external cavity QCL, tunable from 4.385 to 4.58 μm. The tapered fibre and microresonator are kept under a dry and inert atmosphere to preserve them from degradation and physical ageing of the ChG fibre^43^. The QCL light is evanescently coupled to the crystalline microresonator using the ChG tapered fibre. When the taper–resonator coupling is achieved, several resonance families are observed at 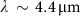, as illustrated in Fig. 2a.

A unique property of taper-coupled microresonators is that phase matching (and thus the coupling) can be finely tuned by translating the microresonator along the taper relative to the waist^33^. Measuring ultra-low losses in microresonators requires an ideal coupler. We investigated in detail the mid-IR coupling behaviour between the ChG taper and the MgF_2_ crystalline microresonator for a typical mode family. For an unity ideality coupler^34^, the coupling parameter is given by 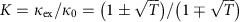, where 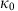 is the intrinsic loss rate, 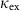 the photon loss rate due to coupling to the microresonator and *T* the transmission on resonance. The upper signs are used for transmission values *T* in the over-coupled regime 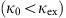 and the lower signs for the under-coupled regime 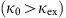. We recorded the transmission spectrum while scanning the laser over resonance for different taper waist radii. We measured the corresponding full-width at half maximum linewidth by setting up a Pound–Drever–Hall technique with a mid-IR electro-optic phase modulator (Qubig GmbH) to provide a frequency calibration. Figure 2c shows the normalized transmission as a function of the coupling parameter *K*. Critical 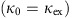 and strong overcoupling up to *K*∼6 are observed for optimum taper diameters. We emphasize that this represents the achievement of critical coupling in crystalline microresonators in the mid-IR region. This reveals that the ChG taper behaves as a nearly ideal coupler, with close to unity ideality^34^. We measured a linewidth of 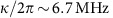 at critical coupling (red curve in Fig. 2b.) yielding an optical factor of *Q*_c_∼1.01 × 10^7^ for MgF_2_. Our measurements reveal that uncoated ChG tapers can thus extend the efficient tapered fibre coupling method deep to the mid-IR regime. It enables an accurate measurement of the material losses in the mid-IR, in contrast to other methods such as prism coupling, which does not allow an ideal coupling^31^,^32^. We point out that it makes very clear that the values are indeed limited by intrinsic multiphonon absorption and not by coupling loss due to lack of ideality. As a consequence, the excellent ability to trace out the coupling curve with high ideality leaves little doubt about the fact that the internal *Q* factor is on the order of 10^7^ and not higher^32^.

### Near- and mid-IR cavity ringdown measurements

**Figure 3 f3:**
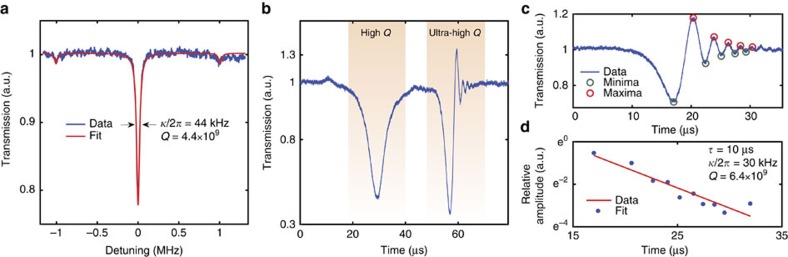
Near-IR characterization of a BaF_2_ microresonator. (**a**) Linewidth measurement (blue line) of an under-coupled resonance with calibration sidebands (resulting from phase modulation of the laser) at 1 MHz and Lorentzian fit (red line). We extract a typical full-width at half maximum of 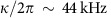 resulting in an optical factor of *Q*∼4.4 × 10^9^. (**b**) Cavity-ringdown measurements. We observed the transmission spectrum of two resonances while scanning at the same speed. Only the ultra-high-*Q* mode features a ringdown signal (right resonance). (**c**) Analysis of ringdown structure. We extract successive amplitudes of maxima (red circles) and of minima (green circles) to fit the evolution of the relative amplitude ringdown (**d**). Theoretical fit of the ringdown relative amplitude (red line). The measured amplitude decay of 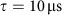 results in a linewidth of 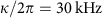 and a *Q* factor of 6.4 × 10^9^.

After MgF_2_ characterization, we studied other fluoride crystals (CaF_2_, BaF_2_ and SrF_2_) and first characterized them in the near-IR set-up. We measured the optical *Q* factor using both frequency modulation spectroscopy and a swept laser cavity ringdown technique. The cavity ringdown method enables a measurement of the quality factor independently of the thermal nonlinearity (hence at higher pumping powers) and of the laser linewidth. Figure 3a shows a typical resonance obtained in the under-coupled regime for a pump power of *P* ≤1 mW in BaF_2_. The Lorentzian fit gives a full-width at half maximum linewidth of 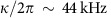 at 1,555 nm, resulting in an optical factor of *Q*∼4.4 × 10^9^. From the coupling curve we can extract moreover an intrinsic linewidth of 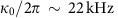 (corresponding to an intrinsic *Q* factor *Q*_0_ of 0.9 × 10^10^). In addition, we performed swept laser cavity ringdown measurements^26^ to first calibrate the method in the near-IR. When an ultra-high-*Q* resonator is excited with a laser whose frequency is linearly swept across the resonance with a duration shorter than the cavity lifetime, its transmission spectrum shows oscillations (Fig. 3b). These oscillations result from the beating of the transiently build-up light inside the resonator that decays into the fibre, with that of the swept laser source. Importantly, when the laser is swept fast, the two components will have a different beat frequency giving rise to an exponentially decaying oscillation. In the case of a lower *Q* mode (with negligible cavity build-up), no ringdown signal is observed at the same scan speed, as highlighted in Fig. 3b. We analysed the transmission spectrum of the ringdown structure in Fig. 3c by measuring the amplitude decay 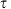 of the remitted light^24^. Its theoretical fit (Fig. 3d) gives a measured amplitude decay 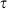 of 10 μs, corresponding to an intrinsic cavity factor 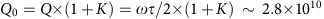 (*Q*∼6.4 × 10^9^). This value is close to the one derived with the frequency modulation spectroscopy, corroborating the method. In CaF_2_ and SrF_2_ microresonators we measured 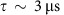 (*Q*∼1.8 × 10^9^), corresponding to an intrinsic cavity factor *Q*_0_∼6.8 × 10^9^, in agreement with the values derived from frequency modulation spectroscopy. We emphasize that we did not resort to annealing procedures to improve *Q* factors of our microresonators^24^.

**Figure 4 f4:**
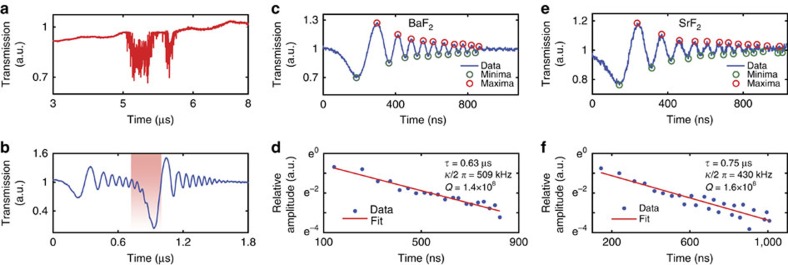
Mid-IR swept frequency ringdowns of BaF_2_ and SrF_2_ microresonators. (**a**) Thermal distortions of a resonance in the BaF_2_ crystalline microresonator. Thermal–optical dynamics prevent measuring the linewidth and the transmission accurately using standard frequency modulation spectroscopy method. (**b**) Using a cavity swept laser ringdown method, we observe modal coupling between two ultra-high-*Q* resonances (red shading). (**c**,**e**) Transmission spectra of the ringdown signal of an ultra-high-*Q* resonance in mid-IR at 4.4 μm using BaF_2_ (**c**) and SrF_2_ (**e**). (**d**,**f**) Exponential fits (red lines) of the minima and maxima data sets (blue circles) extracted from **c**,**e**. In BaF_2_ (**d**), the measured amplitude lifetime of 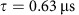 results in a linewidth of 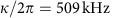 and a corresponding *Q* factor of ∼1.4 × 10^8^. In SrF_2_ (**f**), the measured amplitude lifetime of 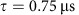 results in a linewidth of 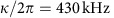 and a corresponding *Q* factor of ∼1.6 × 10^8^.

Having established the ultra-high-*Q* resonance in the near-IR, and having developed the mid-IR ChG tapered fibre method, we afterwards measured *Q* factors in the mid-IR. Phase matching between the ChG tapered fibre and the microresonator is achieved by translating the taper position. In contrast to the near-IR, when coupling is achieved, we observed large thermal instability in the mid-IR^44^. These thermal instabilities induce large distortions of the resonance (Fig. 4a) preventing any reliable measurement of its linewidth^24^,^31^ using the Pound–Drever–Hall error signal as a calibration. In that case, the optical *Q* factor can only be obtained by cavity ringdown measurements. We scanned the QCL using its laser current modulation to observe the cavity ringdown signal. Amplitude and cavity lifetime depend on coupling and linewidth of the resonance. When two resonances are really close, we observe a modal coupling between the two oscillatory decaying signals (Fig. 4b)^45^. The BaF_2_ transmission spectrum of a typical ringdown structure is displayed in Fig. 4c. By measuring the amplitude decay 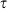 of the remitted light (Fig. 4d), we obtained a BaF_2_ intrinsic *Q* factor of *Q*_0_∼6.2 × 10^8^. CaF_2_ and SrF_2_ crystals were studied by applying the same methods. The ringdown analysis in the case of SrF_2_ is presented in Fig. 4e. We measured an amplitude decay time 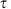 of 0.75 μs (Fig. 4f), resulting in an intrinsic *Q* factor of *Q*_0_∼6 × 10^8^. We note that no ringdown was observed when measuring the *Q* factor of the MgF_2_ microresonator under the same conditions, confirming that MgF_2_ does not feature ultra-high *Q* factors in the mid-IR.

## Discussion

**Figure 5:**
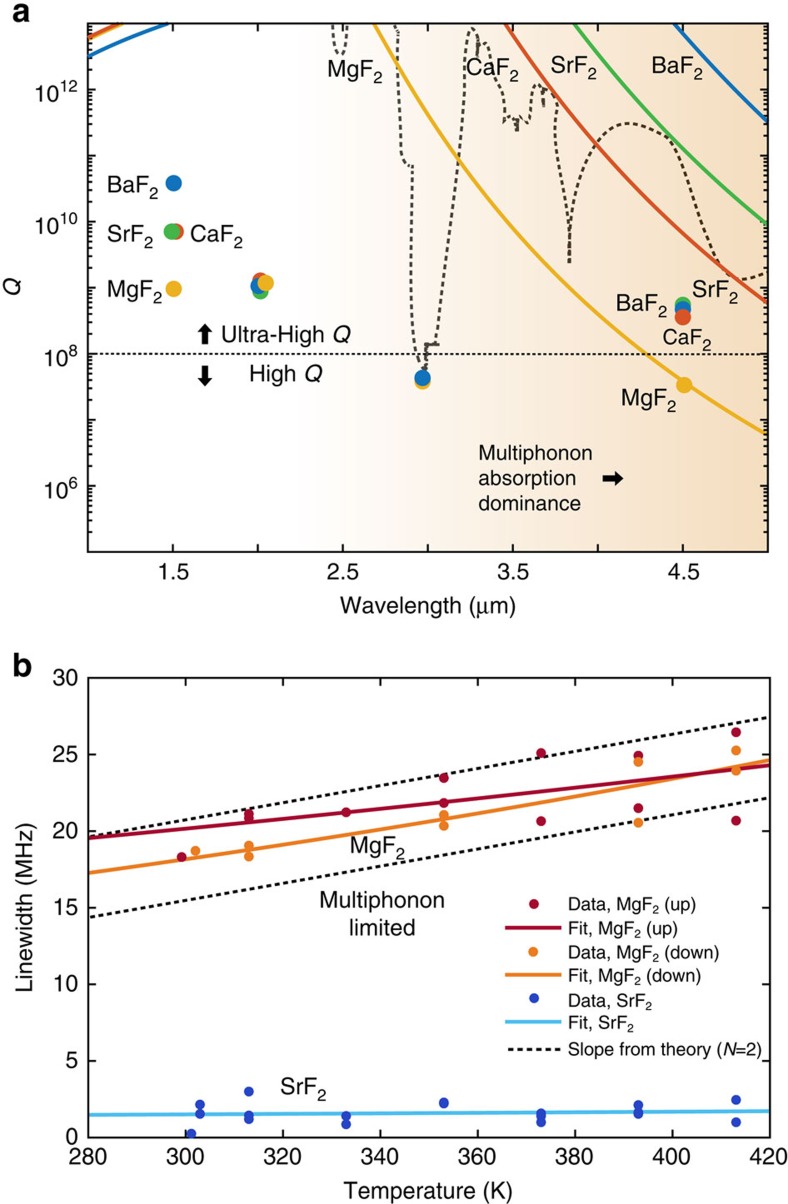
Mid-IR multiphonon absorption in crystalline microcavities. (**a**) Measurements for different fluoride crystals of the *X*F_2_ family (where *X*=Ca, Mg, Ba and Sr) prove the possibility of attaining the ultra-high-*Q* regime in the mid-IR. Except for MgF_2_, for which we reach the theoretical limit imposed by multiphonon absorption (orange shaded region) at room temperature, other materials offer *Q*≥10^8^ around 4.5 μm. The lines represent the theoretical multiphonon absorption limit of *Q* with respect to the wavelength. The circles represent our experimental values. Despite the clear differences with the near-IR region, mid-IR cavities are able to overcome the high-*Q* regime achieving *Q*>10^8^. Measurements around 2 μm show typical level of impurities and defects that limits quality factors when they are not limited by Rayleigh scattering (short wavelengths) or multiphonon absorption (long wavelengths). Measurements around 3 μm highlight that OH absorption can strongly degrade the intrinsic *Q* factor of crystalline materials and constitutes a lower bound of *Q* limitation due to impurities and defects. The dashed grey line is a guide to the eye depicting bulk water absorption *Q* limitation (from ref. 50). Note that 1.5 μm represents the less-affected wavelength by OH absorption (by a few orders of magnitude compared with any other wavelengths). (**b**) Experimental proof of multiphonon absorption in a microresonator made out of an ionic crystal. Temperature sweeps of the microresonator reveal a typical temperature dependence of intrinsic multiphonon absorption for MgF_2_ (fitted slopes of ∼4 × 10^−2^ MHz K^−1^) and consistently no temperature dependence for extrinsic absorption limited SrF_2_ (fitted slope of 10^−3^ MHz K^−1^). Sweep with increasing (up) and decreasing (down) temperatures corroborate the temperature dependence of the intrinsic losses. From the theoretical slope (see Discussion) we infer that two-phonon (*N*=2) processes contribute mostly to the temperature dependence.

The absorption of infrared radiation in the transparency window (absorption level of 10^−3^ cm^−1^ or below) can either be attributed to intrinsic processes involving several phonons or to extrinsic effects involving impurities, defects and surfaces. The difference between intrinsic and extrinsic absorption can be determined by studying the temperature dependence of the absorption. In particular, theory predicts that an exponential temperature dependence is typical of intrinsic multiphonon absorption^35^. The theory considers phonons as bosons, which obey Bose–Einstein occupation of the lattice vibration modes, and thus leads to a dependence of the absorption coefficient in temperature 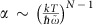 (where the phonon frequency 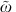 in a *N*-phonon process follows 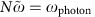). To further verify the intrinsic multiphonon behaviour, we thus measured the quality factor versus temperature by heating the microresonator from room temperature to 420 K for two crystalline materials, for example, MgF_2_ and SrF_2_. The melting of the ChG fibre around 470 K does not allow the temperature to be increased further. For SrF_2_, ringdown measurements show that the *Q* factor value is independent of the temperature, whereas a temperature dependence of the *Q* factor is observed in MgF_2_ (Fig. 5b), confirming that MgF_2_ is limited by multiphonon absorption at 4.5 μm and SrF_2_ is not. From the theoretical slope we infer that two-phonon (*N*=2) processes contribute mostly to the temperature dependence. Extrinsic effects such as impurities can be attributed to the *Q* factor limit of the other crystals. For this purpose, we investigated the *Q* factor at 2 and at 3 μm using a thulium-doped fibre amplifier and an optical parametric oscillator, respectively. Using uncoated ChG tapered fibre and ringdown measurements, we measured similar ultra-high-*Q* factors for all the crystalline materials at 2 μm, corresponding to intrinsic *Q* factors of *Q*_0_≈10^9^. Assigning the origins of the residual extrinsic absorption are delicate due to experimental difficulties and uncertainty of the origins of the absorption themselves. However, we have demonstrated that intrinsic multiphonon absorption limits the *Q* factor of MgF_2_ at 4.5 μm. At very low absorption levels of 10^−4^, surface absorption can also be important. Interestingly, typical internal reflection spectroscopy spectra show strong OH (water) absorption near 3 μm. Our measurements at 3 μm highlight that OH absorption can strongly degrade the *Q* factor of crystalline materials and constitutes a lower bound of the usually flat *Q* limitation due to impurities and defects. The performance of all crystalline materials reveals intrinsic factors of *Q*_0_≈10^7^ near 3 μm. Note that we checked that the decrease of the *Q* factors was not caused by a degradation of the microresonators. For this purpose, they were re-measured at 1,555 nm directly after these new measurements, showing that their ultra-high *Q* factors did not change. Table 1 and Fig. 5 summarize our experimental results. From these measurements we extracted the limiting values of the mid-IR intrinsic absorption 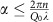 at 4.4 μm. We show that the absorption can thus be as low as 50 p.p.m. per cm for SrF_2_. Translated to a single round trip in a Fabry–Pérot cavity of length *L*/2 (where 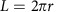 for a WGM of radius *r*) and finesse 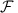, this value amounts to optical losses (absorbance) of the order of 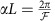, that is, 50 p.p.m.

In summary, we have demonstrated mid-IR ultra-high-*Q* crystalline resonators made from commercially available BaF_2_ and SrF_2_ crystals at 4.5 μm, a region of high interest due to the transparency of the atmosphere and absorption of toxic or greenhouse molecules (for example, CO, CO_2_, SO_2_ or CO_3_) and the availability of QCL sources. Our results in the mid-IR verify the expected temperature variation of the multiphonon losses. Consequently, we show that the intrinsic material absorption in MgF_2_ poses a limit to the *Q* factor, which is overcome in BaF_2_ and SrF_2_. We demonstrate that uncoated ChG tapered fibres can be ideal and efficient couplers deep to the mid-IR. Together with cavity ringdown methods, our platform enables precise measurements of quality factor, overcoming previous limitations. We show that a finesse as high as 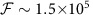 is achievable with BaF_2_ and SrF_2_ in the mid-IR for cavities as small as few millimetres in diameter. This finesse represents more than one order of magnitude improvement over prior high finesse cavities in this wavelength range^3^,^46^,^47^. The exceptional *Q* factor achieved in the mid-IR paves the way for the next generation of ultra-narrow sources and ultra-stable cavities in the molecular fingerprint region and can further leverage QCL technology, by, for example, enabling injection-locked QCL similar to technology developed in the near-IR^10^,^30^. Despite the differences with near-IR, we prove that the mid-IR region is not limited to the high-*Q* regime when proper materials are used. The materials' crystalline nature leads to low thermodynamical noise^21^ and moderate multiphonon absorption in the highly relevant mid-IR region. In addition, combining QCL with mid-IR ultra-high-*Q* crystalline microresonators opens a route for mid-IR Kerr comb or soliton generation^29^,^48^,^49^.

## Methods

### The fabrication of uncoated ChG tapered fibres

To enable repeatable fabrication of high-quality uncoated ChG taper, the physical ageing and instability of ChG tapers must be considered in particular when no coating is used to protect the fibre. A poly(methyl methacrylate) coating provides two key features to subwavelength ChG tapers: high mechanical strength and chemical stability protection. Thus, the fabrication and use of uncoated ChG fibres suffer from high mechanical fragility and strong degradation. When cooling too quickly, an uncoated ChG fibre would break (fast thermal contraction). Adiabatic cooling is used to avoid taper breakage. Tapers are also mounted on a metal mount to strengthen the fibre and keep it stable. Without poly(methyl methacrylate) protection, the physical ageing of ChG glasses is accelerated by exposure to light and in air^43^. Only under controlled inert (Argon) and dry (molecular sieves) atmosphere, will uncoated tapers remain stable as reported in this work. Without these methods, quick ageing of the fibre is observed in just a few days along with degradation of the optical transmission properties.

### Data availability

The data that support the plots within this paper and other findings of this study are available from the corresponding author on reasonable request.

## Additional information

**How to cite this article:** Lecaplain, C. *et al*. Mid-infrared ultra-high-*Q* resonators based on fluoride crystalline materials. *Nat. Commun.* **7,** 13383 doi: 10.1038/ncomms13383 (2016).

**Publisher's note:** Springer Nature remains neutral with regard to jurisdictional claims in published maps and institutional affiliations.

## Supplementary Material

Peer review file

## Figures and Tables

**Table 1:** Measured properties of different crystalline fluoride materials at 4.4 μm.

**Crystal**	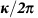	***Q***	***Q*_0_**	α(cm^−1^)	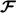
MgF_2_	3.4 MHz	1 × 10^7^	2 × 10^7^	9 × 10^−4^	4 × 10^3^
CaF_2_	710 kHz	9.6 × 10^7^	3.5 × 10^8^	5 × 10^−5^	7.8 × 10^4^
BaF_2_	509 kHz	1.4 × 10^8^	6.2 × 10^8^	3.3 × 10^−5^	1.3 × 10^5^
SrF_2_	430 kHz	1.6 × 10^8^	6 × 10^8^	3.2 × 10^−5^	1.5 × 10^5^
