# Supplementary material for: Mid-infrared ultra-high-Q resonators based on fluoride crystalline materials
Source: Nat Commun. 2016 Nov 21;7:13383. doi: 10.1038/ncomms13383 (PMC5121327; doi:10.1038/ncomms13383)
Supplement: Peer review file [file ncomms13383-s1.pdf]

Editorial Note: this manuscript has been previously reviewed at another journal that is not operating a transparent peer review scheme. This document only contains reviewer comments and rebuttal letters for versions considered at Nature Communications.
